# Quantitative Assessment of Bilateral Upper-Limb Kinematics During Activities of Daily Living Using an Optoelectronic Motion-Capture System

**DOI:** 10.3390/s26216747

**Published:** 2026-10-25

**Authors:** Filipe Galhanas Sá, Patrícia Santos, Carla Quintão, Cláudia Quaresma

**Affiliations:** 1 Laboratory for Instrumentation, Biomedical Engineering and Radiation Physics (LIBPhys), NOVA School of Science and Technology, NOVA University of Lisbon, 2829-516 Caparica, Portugal; par.santos@campus.fct.unl.pt (P.S.); cmquintao@fct.unl.pt (C.Q.); q.claudia@fct.unl.pt (C.Q.); 2 Associated Laboratory in Translation and Innovation Towards Global Health (REAL), NOVA School of Science and Technology, NOVA University of Lisbon, 2829-516 Caparica, Portugal; 3 Physics Department, NOVA School of Science and Technology, NOVA University of Lisbon, 2829-516 Caparica, Portugal; 4 Health Department, Polytechnic University of Beja, 7800-111 Beja, Portugal

**Keywords:** activities of daily living, upper limb, kinematics, optoelectronic motion capture, three-dimensional motion analysis, functional movement assessment

## Abstract

Background: Quantitative assessment of upper-limb movement during activities of daily living may complement conventional clinical scales and provide objective information on joint coordination. Methods: Three-dimensional upper-limb kinematics were recorded in 38 healthy right-handed adults using a four-camera Qualisys optoelectronic motion-capture system operating at 100 Hz. Thirty-six reflective markers were used to model the trunk, upper arms, forearms, and hands. Participants performed five repetitions of eating and cutting tasks. Following quality control, data from 33 participants were analyzed for eating and data from 28 participants for cutting. Joint-angle trajectories were time-normalized and descriptively analyzed for both upper limbs. Results: During eating, the dominant elbow presented the largest flexion–extension range of motion, 55 ± 22°, followed by shoulder flexion–extension, 34 ± 17°, and wrist flexion–extension, 21 ± 12°. During cutting, elbow flexion–extension showed the largest range of motion in both the dominant and non-dominant limbs, 63 ± 18° and 61 ± 18°, respectively. Pronation–supination exhibited marked between-participant variability. Conclusions: Optoelectronic motion capture enabled the characterization of bilateral upper-limb kinematics across predefined phases of functional tasks, while joint-angle ranges of motion were quantified over the complete activity. The results provide preliminary reference patterns and support further studies comparing healthy participants with individuals with neurological or musculoskeletal impairments.

## 1. Introduction

Neurological disorders, such as stroke, Parkinson’s disease, and multiple sclerosis, frequently lead to impairments in upper-limb (UL) function, significantly affecting an individual’s ability to perform Activities of Daily Living (ADL) [1,2]. These impairments may involve motor, sensory, and coordination deficits, often resulting in reduced independence and quality of life. Consequently, rehabilitation aims to restore functional capacity and improve performance in everyday tasks [1].

ADL, such as self-feeding or food preparation, require complex and coordinated movements of the upper limbs [3]. These activities may involve unilateral and bilateral actions, object manipulation, and sequential movements, placing high demands on motor control and inter-joint coordination [4]. Given this complexity, accurate assessment of UL function is essential for understanding movement limitations and guiding rehabilitation.

In clinical practice, functional assessment is commonly based on ordinal rating scales, such as the Barthel Index (BI) [5]. Although widely used, these tools provide only a coarse evaluation of performance and lack sensitivity to detect subtle but clinically relevant changes. Patients may demonstrate improvements in movement quality or coordination that are not reflected in their scores [5,6]. Additionally, compensatory strategies—particularly involving the non-affected limb—may go unnoticed, potentially leading to inefficient or maladaptive movement patterns over time.

To overcome these limitations, objective and quantitative methods have been increasingly explored. Kinematic analysis enables a detailed characterization of movement by examining joint angles, temporal patterns, and coordination between segments [7,8]. However, most existing studies have focused on simplified tasks, such as drinking, and have typically analyzed only one limb, failing to capture the complexity of real-world ADL that require bilateral coordination [9,10,11]. Although upper-limb kinematics have been extensively investigated during reaching and drinking, fewer studies have characterized bilateral joint-angle trajectories during multistep eating and food-cutting tasks. Moreover, the kinematic contribution of the non-dominant limb during tasks conventionally classified as unilateral remains insufficiently described.

Furthermore, the interpretation of kinematic data in clinical populations depends on the availability of normative reference patterns obtained from healthy individuals. Without these benchmarks, it is difficult to distinguish pathological movement patterns from normal variability in motor strategies [12,13]. Despite this need, there is still limited information regarding upper-limb kinematics during functional, bilateral ADL with different motor demands, such as eating and cutting.

Therefore, the present study aimed to characterize the bilateral three-dimensional kinematics of the shoulder, elbow, forearm, and wrist during two representative and personally meaningful activities of daily living: eating and cutting. Specifically, we sought to characterize joint-angle trajectories across predefined task phases, quantify joint-angle ranges of motion over the complete activity, and examine the contribution of the dominant and non-dominant limbs in healthy right-handed adults using an optoelectronic motion-capture system. The resulting reference patterns may support more objective assessment of upper-limb function and provide a basis for future comparisons with individuals with neurological impairments.

## 2. Materials and Methods

### 2.1. Participants

A convenience sample of 38 healthy right-handed individuals participated in this study (14 males, 24 females; age: 30 ± 12 years; height: 1.68 ± 0.11 m; body mass: 64 ± 11 kg). Demographic and anthropometric information, including hand dominance, was obtained through a questionnaire completed by the participants.

The following were considered exclusion criteria for participants: a diagnosis of neuromuscular, cognitive, or language deficits, as well as visual acuity impairments not corrected by glasses or contact lenses.

Participation was voluntary, and all participants provided written informed consent in accordance with the Declaration of Helsinki. Ethical approval was obtained from the Ethics Committee of the NOVA School of Science and Technology—NOVA FCT.

### 2.2. Data Collection Procedures

**Figure 1:**
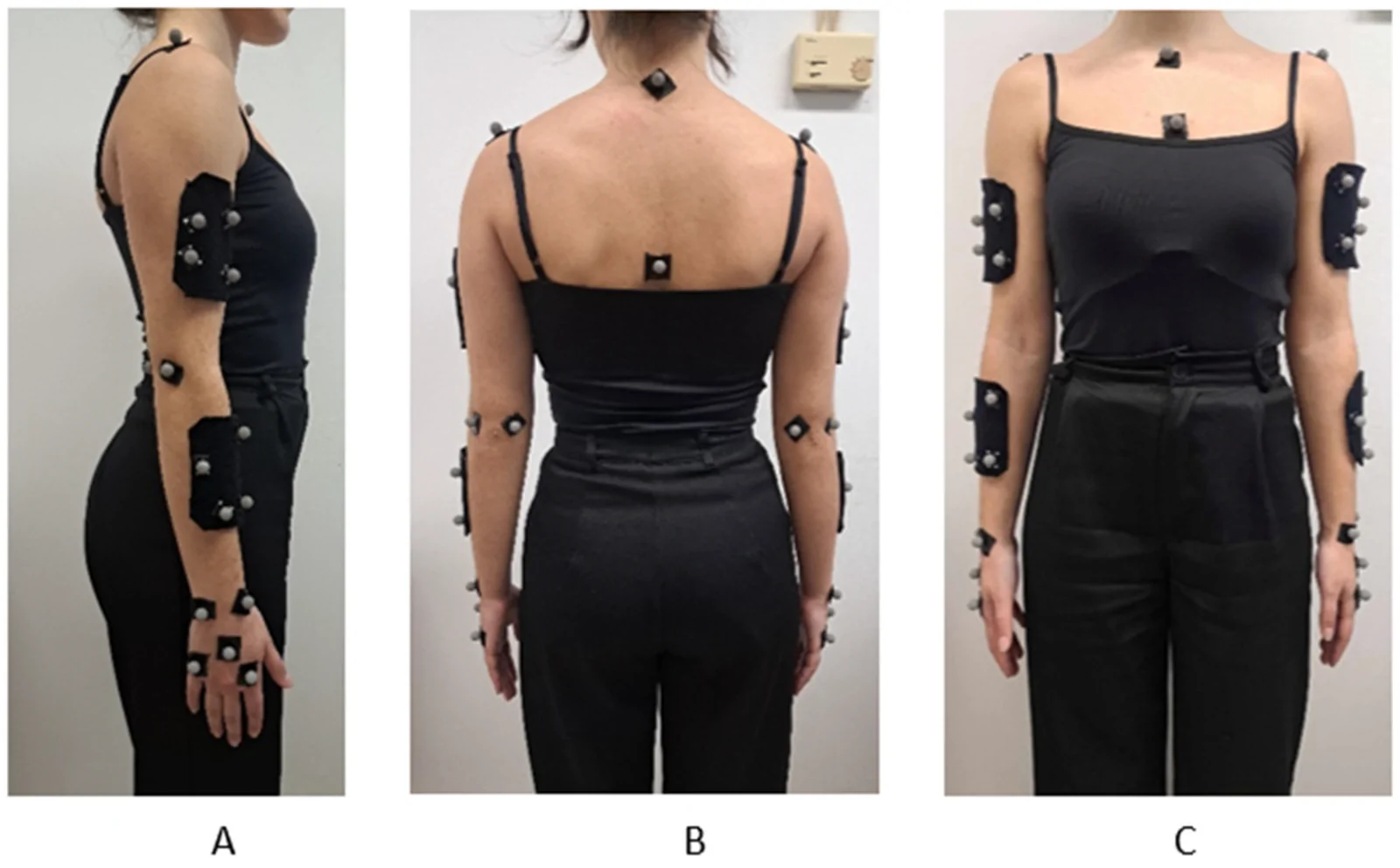
Reflective markers placed on one participant from the sample, shown from lateral (**A**), posterior (**B**) and anterior (**C**) views.

The Qualisys Track Manager (QTM) software is a kinematic data acquisition platform that enables the accurate capture of three-dimensional motion. In this study, QTM software^®^ (version 2024.2, Qualisys AB, Gothenburg, Sweden) was used in conjunction with a Qualisys^®^ optoelectronic measurement system (Qualisys^®^, Gothenburg, Sweden), comprising four infrared cameras (Miqus model) that tracked the trajectories of 36 reflective markers placed at specific anatomical positions, with a sampling frequency of 100 Hz [14]. Sixteen of these markers were arranged in clusters of four, used to track the movement dynamics of each forearm and arm, while the remaining 20 markers were placed individually. In each participant, the markers were affixed to the skin using double-sided adhesive tape. The anatomical locations of all markers, along with the abbreviations of their respective names, are detailed in Table 1. In addition, Figure 1 provides lateral (A), posterior (B) and anterior (C) views of the participants with all markers in place.

The reflective markers were placed with the aim of enabling the subsequent construction of a skeletal model comprising the trunk, upper arm, forearm, and hand [15].

Regarding the execution of the activities, the protocol followed was adapted from a study conducted by Santos et al. [16,17,18], which, although focused on the analysis of electrical activity, shares the same ADL investigated in the present study.

**Figure 2:**
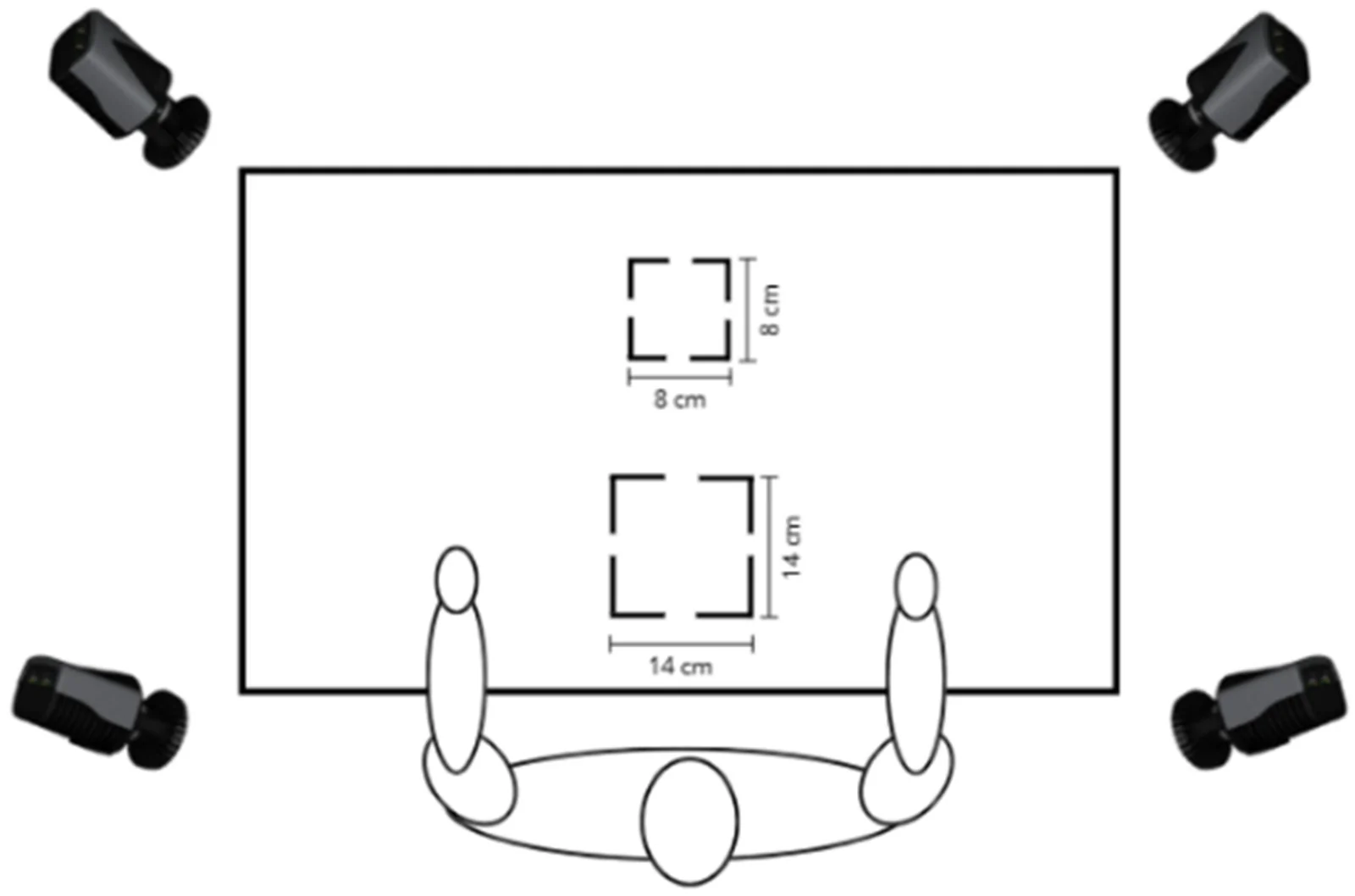
Experimental set-up.

After the participants were prepared for data collection, the procedure adopted consisted of the following [16,17,18]:

- Participants first performed a midline directed activity (eating soup) with the dominant limb, followed by a bilateral activity (cutting), during which the dominant limb manipulated the knife and the non-dominant limb stabilized the fork.
- During the activities, the subjects were seated in a chair without an armrest (40 cm high) with the dorso-lumbar region of the spine supported on the back of the chair, next to a table (75 cm height), with the feet well supported on the floor, knees and hips flexed at 90°. The upper limbs were placed on the table, assuming as the initial position the shoulder in neutral position, elbow flexed at 90°, forearm in pronation wrist in neutral position and fingers in extension.
- The experimental setup is illustrated in Figure 2. The materials were placed before the start of the rehearsal of each of the respective activities, in the respective marks on the table towards the midline of the subjects’ body: the cup at the 8 × 8 cm mark 30 cm from the edge of the table and the plate at the 14 × 14 cm mark at 3 cm from the edge of the table, with the knife and fork placed side by side beside the plate.
- Before data collection, each activity was explained and given the opportunity to perform the movement so that subjects feel comfortable with the execution of it. Participants were instructed to perform five trials for each of the two activities, resulting in a total of 10 trials, following the protocol adopted in previous studies by Santos et al. [16,17,18].
- Once the subjects are ready, the command “you can start now” is given and the subject performed the activity at a speed comfortable for him/herself and five trials were collected.
- Between each trial, subjects had a 5 s rest period and a further rest period (5 min) between both activities to minimize muscle fatigue.
- After completing the performance of the ADL, a static position was recorded for each participant using the kinematic data acquisition system. This position was selected to ensure that all markers were visible for at least one second, and in most cases corresponded to the anatomical position, with the forearms in pronation.

### 2.3. Division of Activities into Phases

Similar to what has been done in previous studies [11,16,17,18], it has become necessary to divide activities into different phases, as they involve a series of coordinated movements between multiple joints. Thus, it was possible to divide the eating activity into seven phases (1: initial position to reach, 2: grasping, 3: transport to mouth, 4: introduced into mouth, 5: return to table, 6: return to initial position, and 7: rest), and the cutting activity into eight phases (1: initial position to reach, 2: grasping, 3: manipulate, 4: transport to the mouth, 5: introduced into mouth, 6: return to table, 7: return to initial position, and 8: rest), as shown in Table 2.

The first author of the article identified the phases of movement through visual inspection of frontal-view video recordings. To this end, the videos were first temporally aligned with the kinematic data acquired by the Qualisys^®^ system, using a synchronization procedure based on a common reference event, which consisted of a brief mechanical impact applied to a reflective marker visible to the infrared cameras and simultaneously detectable in the video recordings. This event allowed precise temporal correspondence between the video frames and the kinematic data.

After synchronization, each video was cropped from the reference point and analyzed frame by frame in MATLAB^®^ (R2025b, MathWorks, Natick, MA, USA), using a custom-developed function to record the frames corresponding to the start and end of each phase for the selected movement cycles.

Phase identification was performed by a single observer. Although no formal intra- or inter-observer reliability analysis was conducted, predefined criteria based on objectively observable movement transitions were consistently applied across all participants and trials. These transitions corresponded to specific events defining the beginning and end of each phase, with the end of each phase coinciding with the beginning of the subsequent phase. For Phase 1, the initial time point was defined as the moment immediately preceding the occurrence of the participant’s first observable movement, corresponding to the effective onset of the activity. Thus, phase delineation followed a continuous and sequential structure throughout each activity cycle. In cases of uncertainty regarding the identification of a specific transition, sagittal-plane video recordings were also consulted to support phase delineation and confirm the occurrence of specific movement events.

MATLAB^®^ (R2025b, MathWorks, Natick, MA, USA), was employed to analyze the frontal .mov video files frame by frame, using a function that recorded in an Excel file the specific frame at which each phase began and ended. Based on the frame count and the camera sampling rate (30 Hz), the time (in seconds) for each phase was determined, and the mean and standard deviation (SD) of the duration of each phase were calculated.

For data presentation, the mean duration of each phase was used to time-normalize the movement cycle to 100%, allowing each phase to be expressed as a percentage of the total cycle. This normalization allowed the presentation of the movement cycle (average of five repetitions) as a single complete cycle representing 100% of the time, with the phases superimposed on the joint angle values to provide a clearer visualization of movement coordination throughout the task.

The calculated phase durations were used exclusively for temporal normalization of the movement cycle and to define the phase boundaries displayed in the joint-angle profiles. As phase duration was not one of the study outcomes, these values are not presented separately in the Section 3.

### 2.4. Data Processing

Once the data collection phase was completed, the data obtained was pre-processed using the QTM software. The dataset included one static acquisition and two dynamic acquisitions (one for each activity) for all subjects in the sample. The trajectories of each marker were identified, and the continuity of these trajectories throughout the acquisition period of each activity was verified for all subjects. This process was carried out separately for each ADL, with the aim of ensuring that, in every activity, all body segments to be analyzed contained at least three tracking markers with a fill level of at least 98% [15].

To ensure data quality (fill level = 98%), it was therefore necessary to fill the gaps using different interpolation methods provided by the QTM software. The Linear Method was used for short gaps, allowing simple interpolation between the ends of the trajectory, with the possibility of extrapolation in cases where the gap occurred at the beginning or end of the acquisition. Polynomial filling, based on cubic interpolation, was applied when data existed before and after the gap, providing a smoother transition between segments. The Relational Method was used exclusively in more complex cases, especially in regions with marker clusters. In these situations, the missing trajectory was reconstructed based on the movement of adjacent markers and the analysis of local geometry, allowing for a more accurate reconstruction.

During this stage of the study, it was found that some participants, during certain ADLs, had gaps in the data that could not be reconstructed, possibly due to temporary calibration issues with the Qualisys motion capture system. Consequently, participants were excluded only from the analyses of the activities for which complete kinematic data were not available. Therefore, the effective sample size differed between activities, resulting in 33 participants for the eating task and 28 participants for the cutting task, from an initial sample of 38 participants [14].

Following this stage, the marker trajectory data were exported to Visual3D^®^ (version 2023.08.3, HAS-Motion, Kingston, ON, Canada) in a format compatible with the software for further processing and analysis.

Within Visual3D, a biomechanical model of the trunk and upper limbs was developed based on the software’s official guidelines and adapted from the model proposed by Pedro et al. [15]. To define a body segment within the software, at least three non-collinear tracking markers are required, enabling the establishment of a rigid local coordinate system. In addition, the proximal and distal endpoints of each segment must be identified using either anatomical markers, placed on palpable bony landmarks during the static acquisition, or virtual landmarks generated within Visual3D^®^ (version 2023.08.3, HAS-Motion, Kingston, ON, Canada) based on the geometry of the physical markers.

Prior to model construction, ten landmarks were defined to enable the identification of the different body segments. Subsequently, the static acquisition file of one participant was selected and used to develop the three-dimensional model. The body segments, considered rigid bodies, were defined according to the position of the reflective markers and corresponding landmarks, resulting in the delineation of seven segments: trunk, left arm, right arm, left forearm, right forearm, left hand, and right hand. An orthogonal Cartesian coordinate system was assigned to each segment according to the methodology described by Kim et al. [11]. Considering the anatomical position as the reference posture, the *X*-axis corresponded to the lateral (+) and medial (−) direction, the *Y*-axis to the anterior (+) and posterior (−) direction, and the *Z*-axis to the proximal (+) and distal (−) direction. For the upper limbs, the *X*-axis orientation differed between sides: on the right side, it was oriented from medial to lateral (+), whereas on the left side it was oriented from lateral to medial (+).

The model was subsequently saved in .mdh format and individually applied to the static files of all participants. The association between each participant’s static and dynamic trials enabled the automatic use of the model for segment reconstruction during the analysed activities.

Subsequently, and still within the Visual3D^®^ (version 2023.08.3, HAS-Motion, Kingston, ON, Canada) software, joint angles were calculated based on the methodology described by Kim et al. [11]. The rotation order of the distal segment relative to the proximal segment followed each segment’s local coordinate system using an X–Z–Y Cardan sequence, to align with clinical terminology. In the coordinate axes used, the X, Y, and Z axes correspond to the lateral–medial, anterior–posterior, and vertical directions, respectively. This methodology allowed, for both upper limbs, the calculation of shoulder flexion (+)/extension (−), abduction (+)/adduction (−), and medial (+)/lateral (−) rotation; elbow flexion (+)/extension (−) and pronation (+)/supination (−); and wrist flexion (+)/extension (−).

After extracting the joint angles, a moving average with a 1-s window was applied to the raw signal in MATLAB^®^ (R2025b, MathWorks, Natick, MA, USA) to attenuate high-frequency variations. The window was defined in the time domain, rather than as a fixed number of samples, ensuring that the smoothing parameter remained independent of the acquisition frequency. This approach reduced short-term fluctuations while preserving the movement dynamics at the timescale of seconds, which was considered appropriate for characterising the movement.

Each activity was divided into five individual cycles and, for each cycle, the joint angle data were corrected by subtracting the mean value corresponding to the final resting phase included in that activity. Consequently, the angles presented do not correspond to absolute anatomical angles, but rather to angular changes relative to each participant’s resting posture. The final resting phase was used as a reference as it was the only point in time common to all activities and all cycles.

To enable meaningful comparisons between participants, the corrected joint angle cycles were temporally interpolated to a common duration, as each participant performed the activities over different time periods. This temporal normalisation ensured that equivalent phases of movement were aligned across participants, allowing for point-by-point comparison of joint angle profiles and the calculation of representative average curves [19].

Subsequently, the mean of the cycles per participant was calculated, and these individual means were aggregated into a matrix for collective analysis, from which the mean behavior per joint across all participants was extracted.

Once the mean profiles for each joint had been obtained, descriptive statistics were calculated, specifically the mean and standard deviation of the kinematic variables analysed. No inferential statistical analyses were performed; consequently, no formal comparisons were made between activities, between the dominant and non-dominant limbs, nor was a statistical assessment of inter-individual variability carried out.

## 3. Results

As in previous studies [9,10,11], the maximum and minimum joint angles, as well as the range of motion (RoM) values of the upper-limb joints evaluated in this study, were obtained and are presented in Table 3 for the eating activity and in Table 4 for the cutting activity.

**Figure 3:**
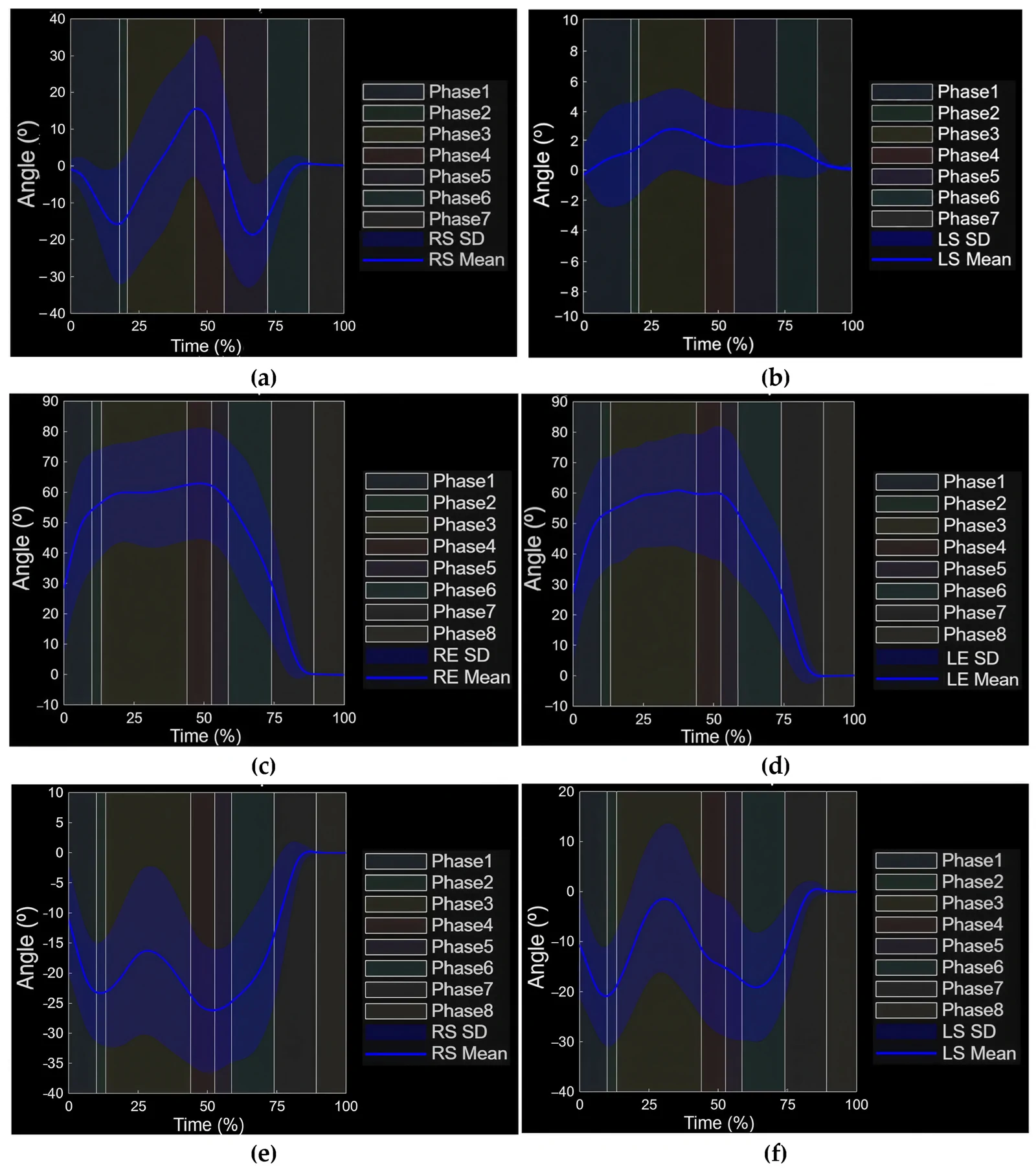
Dominant (**a**), non-dominant (**b**) shoulder flexion (+)/extension (−) and dominant (**c**), non-dominant (**d**) elbow flexion (+)/extension (−) during the ADL of eating. Dominant (**e**), non-dominant (**f**) shoulder flexion (+)/extension (−) during the ADL of cutting. The blue lines and shaded areas indicate the mean and standard deviation of the joint angles, respectively. The vertical phase boundaries correspond to the mean duration of each movement phase across participants after temporal normalization of the movement cycle.

In addition, the temporally normalized joint-angle profiles for the evaluated joints are presented in Figure 3. The blue lines represent the mean joint-angle trajectories across participants, while the shaded areas indicate the corresponding standard deviations. The boundaries between movement phases correspond to the mean duration calculated for each phase across participants, allowing direct comparison of joint-angle trajectories throughout task execution.

Regarding the range of pronation/supination movement, the eating task exhibited greater variability in the dominant elbow, as reflected by the higher standard deviation (SD). In contrast, during the cutting task, greater variability was observed in the non-dominant limb. This variability may reflect differences in the motor strategies adopted by participants while performing these tasks.

All comparisons presented in this section are descriptive and should not be interpreted as evidence of statistically significant differences between activities or limbs, as no inferential statistical analyses were performed.

## 4. Discussion

The eating task requires specific motor skills involving the shoulder, elbow, and wrist of the dominant upper limb, demanding precise coordination between the different upper-limb segments. This movement pattern may reflect an integration of proximal and distal movements, with proximal movements contributing to the overall positioning of the limb and distal movements supporting the orientation and manipulation of the spoon throughout the different phases of the task.

At the shoulder, the greatest range of motion was observed in flexion/extension, reflecting the need to elevate the spoon toward the mouth and subsequently return it to the table through smooth and coordinated movements. The kinematic profile shown in Figure 3a demonstrates a progressive increase in shoulder flexion during the spoon transport phase, reaching its maximum immediately before food intake, followed by a gradual decrease (extension) as the upper limb returns to its initial position. This behavior may reflect an important contribution of the shoulder to the positioning of the upper limb throughout the task.

At the elbow, the flexion/extension range of motion (55 ± 22°) was the largest among all joint movements. This finding suggests a substantial contribution of the elbow to bringing the spoon closer to the body, particularly during the initial reaching phase, alongside the observed contribution of the shoulder throughout the movement. Regarding forearm pronation/supination, the eating task exhibited a considerable range of motion accompanied by high variability (SD = 35°).

Unlike shoulder flexion/extension, which showed a highly consistent pattern across participants, forearm pronation/supination demonstrated substantially greater variability, suggesting that forearm orientation allows greater flexibility in task execution strategies. This finding became evident after individual inspection of each participant’s joint-angle profiles before calculating the mean trajectories. Therefore, the high standard deviations observed may reflect physiological variability in movement execution. However, confirmation of this hypothesis requires a more comprehensive quantitative analysis and a larger sample size, which should be addressed in future studies, particularly in the development of normative movement databases.

Comparison of Figure 3a–d reveals that, contrary to what might be expected, shoulder flexion is primarily associated with the food intake phase, corresponding to transporting the spoon toward the mouth, whereas the elbow plays a more prominent role during the initial reaching phase from the resting position. This complementary pattern may suggest distinct functional contributions of the shoulder and elbow across the different phases of the movement. However, these observations should be interpreted descriptively, as no formal analysis of inter-joint coordination was performed.

At the wrist, the flexion/extension range of motion was 21 ± 12°. Although smaller than that observed at the shoulder and elbow, this result suggests a contribution of the wrist to spoon manipulation and fine motor control, potentially supporting the orientation adjustments required during food intake.

Regarding the non-dominant upper limb, although it was not directly involved in manipulating the spoon, small variations in joint amplitude were observed, corresponding to subtle postural adjustments performed instinctively by the participants. Specifically, the non-dominant shoulder flexion/extension profile (Figure 3b) showed small oscillations that occurred predominantly immediately before the phases in which the dominant limb performed its largest movements, particularly during spoon transport toward the mouth. These findings may suggest a stabilizing contribution of the non-dominant limb during this unilateral task, potentially reflecting postural adjustments accompanying the movements of the dominant limb.

The cutting task involves movements of both upper limbs, with the dominant and non-dominant limbs performing different and apparently complementary functions. While the dominant limb manipulates the knife and generates the cutting motion, the non-dominant limb stabilizes the food using the fork, ensuring the precision and stability required to successfully complete the task.

At the shoulder, the dominant limb exhibited a flexion/extension range of motion of 26 ± 9°, whereas abduction/adduction movements remained limited in both upper limbs. These findings suggest that, once the cutlery has been positioned, the shoulder may contribute primarily to maintaining the overall positioning of the upper limb, with relatively small adjustments throughout task execution. In contrast, medial/lateral rotation showed a greater range of motion in the non-dominant limb (24 ± 12°), reflecting the continuous adjustment of fork orientation required to stabilize the food during cutting.

Comparison of the dominant and non-dominant shoulder flexion/extension profiles (Figure 3e,f) shows a similar temporal evolution of both shoulders throughout the task, which may suggest a related temporal movement pattern despite markedly different movement amplitudes. In both limbs, the progressive increase in shoulder flexion (positive slope) occurs during the manipulation phase and the positioning required for cutting. However, the dominant shoulder exhibits greater amplitudes, which may be related to the demands associated with knife manipulation, whereas the non-dominant shoulder shows smaller adjustments that may be related to fork positioning and food stabilisation. Conversely, during the transport of the cutlery toward the mouth, both shoulders display a negative slope, corresponding to shoulder extension. This behavior may suggest a reduced contribution of shoulder movement during this phase, while the final approach of the cutlery to the mouth is accompanied by increased elbow flexion. This pattern may indicate a greater contribution of elbow movement during food insertion.

The elbow exhibited the greatest range of motion among all joints, with flexion/extension values exceeding 60° in both upper limbs, suggesting a substantial contribution of elbow movement to the execution of the cutting task. Unlike the eating task, where elbow flexion/extension is prominent during the reaching phase, the cutting task showed repeated elbow flexion and extension during the manipulation phase, which may reflect an important contribution of the elbow to the cyclic knife movement.

Regarding forearm pronation/supination, the non-dominant limb exhibited a particularly high standard deviation (54°), once again suggesting considerable inter-individual variability. 

At the wrist, flexion/extension amplitudes were relatively small but may nevertheless reflect a contribution to the fine adjustment of knife orientation during the cutting task.

Several limitations should be considered when interpreting the findings of this study. First, the use of a convenience sample of healthy right-handed participants limits the generalizability of the results to broader populations. Although 38 participants were initially recruited, the final sample size differed between activities because participants with incomplete kinematic data were excluded only from the affected activity, resulting in 33 participants for the eating task and 28 for the cutting task. This difference in sample size should therefore be considered when interpreting the descriptive findings.

Second, movement-phase identification was performed by a single observer based on visual inspection of synchronized video recordings. Although predefined criteria based on observable movement transitions were consistently applied, no formal intra- or inter-observer reliability analysis was performed. Consequently, the potential influence of observer-related variability on phase delineation cannot be excluded.

Finally, the analysis was intentionally descriptive, and no formal inferential statistical analyses were performed.

Future studies should include larger and more representative samples, multiple observers or formal reliability assessment, measurement-error analyses, and appropriate inferential statistical methods.

## 5. Conclusions

This study used three-dimensional optoelectronic motion capture to characterize bilateral upper-limb kinematics during eating and cutting tasks in healthy right-handed adults. The descriptive findings showed that elbow flexion–extension exhibited the largest range of motion in both activities, while the shoulder appeared to contribute mainly to limb positioning and spatial orientation and the wrist to the fine adjustments required for utensil manipulation.

The simultaneous analysis of both upper limbs also revealed substantial bilateral involvement during the cutting task and small angular excursions of the non-dominant limb during eating. These movements may reflect postural adjustments and associated bilateral coordination, although their functional significance should be interpreted cautiously in the absence of formal statistical comparisons and measurement-error analyses. Forearm pronation–supination showed considerable between-participant variability, particularly in the dominant limb during eating and in the non-dominant limb during cutting. Further investigation is required to determine whether this variability reflects differences in motor strategies, methodological factors, or both.

Overall, the findings provide preliminary reference patterns for the bilateral assessment of upper-limb movement during functional tasks. These results may support the design of future studies involving individuals with neurological or musculoskeletal impairments and contribute to the development of more objective and sensitive approaches to functional movement assessment.

## Figures and Tables

**Table 1:** Marker names and corresponding anatomical locations.

Marker Names	Anatomical Location
C7	On the spinous process of the 7th cervical vertebra
T10	On the spinous process of the 10th thoracic vertebra
STR_UP	On the jugular notch
STR_DOWN	On the body of the sternum
LAC	On the left acromion
RAC	On the right acromion
LLELBOW	On the left lateral epicondyle
RLELBOW	On the right lateral epicondyle
LMEELBOW	On the left medial epicondyle
RMEELBOW	On the right medial epicondyle
LLATWRIST	On the left radial styloid process
RLATWRIST	On the right radial styloid process
LMEDWRIST	On the left ulnar styloid process
RMEDWRIST	On the right ulnar styloid process
L3MET	On the center of the 3rd metacarpal of the left hand
R3MET	On the center of the 3rd metacarpal of the right hand
L2MET	On the distal end of the 2nd metacarpal of the left hand
R2MET	On the distal end of the 2nd metacarpal of the right hand
L5MET	On the distal end of the 5th metacarpal of the left hand
R5MET	On the distal end of the 5th metacarpal of the right hand
LUPARM1,2,3,4 (cluster)	On the anterolateral region of the left upper arm
RUPARM1,2,3,4 (cluster)	On the anterolateral region of the right upper arm
LARM1,2,3,4 (cluster)	On the anterolateral region of the left forearm
RARM1,2,3,4 (cluster)	On the anterolateral region of the right forearm

**Table 2:** Activity phases in cutting activity.

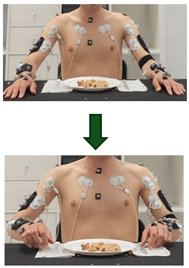	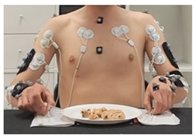	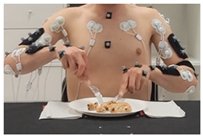	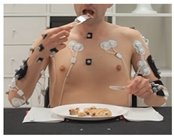
**Phase 1: Initial Position to Reach**	**Phase 2: Grasping**	**Phase 3: Manipulation**	**Phase 4: Transport to the Mouth**
** 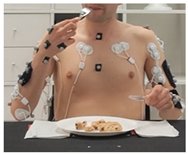 **	** 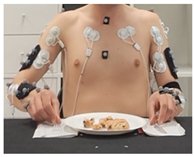 **	** 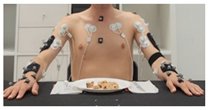 **	…
**Phase 5: Introduced into Mouth**	**Phase 6: Return to the Table**	**Phase 7: Return to the Initial Position**	**Phase 8: Rest**

**Table 3:** Joint angles analyzed during phases of eating task for both limbs. Mean (standard deviation). Note: values presented relative to the resting position.

Kinematic Variables	Maximum Joint Angle (deg)		Minimum Joint Angle (deg)		Range of Motion (deg)	
	Dominant Limb	Non-Dominant Limb	Dominant Limb	Non-Dominant Limb	Dominant Limb	Non-Dominant Limb
*Shoulder* Flexion–extension	16 ± 19	3 ± 3	−19 ± 14	−1 ± 2	34 ± 17	3 ± 3
Abduction–adduction	11 ± 9	1 ± 2	0 ± 0	0 ± 1	11 ± 6	1 ± 2
Medial–lateral rotation	3 ± 10	2 ± 2	−10 ± 8	−1 ± 12	13 ± 9	3 ± 11
*Elbow* Flexion–extension	54 ± 19	0 ± 5	−1 ± 4	−1 ± 5	55 ± 22	2 ± 7
Pronation–supination	2 ± 17	2 ± 7	−32 ± 27	−1 ± 3	34 ± 35	3 ± 12
*Wrist* Flexion–extension	15 ± 18	0 ± 1	−6 ± 16	0 ± 1	21 ± 12	1 ± 2

**Table 4:** Joint angles analyzed during phases of cutting task for both limbs. Mean (standard deviation). Note: values presented relative to the resting position.

Kinematic Variables	Maximum Joint Angle (deg)		Minimum Joint Angle (deg)		Range of Motion (deg)	
	Dominant Limb	Non-Dominant Limb	Dominant Limb	Non-Dominant Limb	Dominant Limb	Non-Dominant Limb
*Shoulder* Flexion–extension	0 ± 1	1 ± 1	−26 ± 10	−21 ± 10	26 ± 9	21 ± 8
Abduction–adduction	8 ± 9	9 ± 7	0 ± 0	0 ± 0	8 ± 6	9 ± 6
Medial–lateral rotation	4 ± 12	14 ± 14	−14 ± 7	−11 ± 7	18 ± 8	24 ± 12
*Elbow* Flexion–extension	63 ± 18	61 ± 19	0 ± 0	0 ± 1	63 ± 18	61 ± 18
Pronation–supination	3 ± 23	6 ± 28	−6 ± 23	−17 ± 31	10 ± 24	23 ± 54
*Wrist* Flexion–extension	6 ± 11	9 ± 13	−5 ± 10	−7 ± 15	11 ± 7	16 ± 6

## Data Availability

The original contributions presented in this study are included in the article. Further inquiries can be directed to the corresponding author.
